# Integrated Green Valorization of Coffee Cherry Pulp Through Bioactive Compound and Pectin-Rich Fraction Recovery for Pectic Oligosaccharide Production

**DOI:** 10.3390/foods15193555

**Published:** 2026-10-06

**Authors:** Kamon Yakul, Tippapha Pisithkul, Charin Techapun, Chayatip Insomphun, Churairat Moukamnerd, Phisit Seesuriyachan, Wilasinee Jirarat, Thanongsak Chaiyaso

**Affiliations:** 1 Division of Biotechnology, Faculty of Agro-Industry, Chiang Mai University, Chiang Mai 50100, Thailand; kamon.y@cmu.ac.th (K.Y.); charin.t@cmu.ac.th (C.T.); chayatip.i@cmu.ac.th (C.I.); churairat.m@cmu.ac.th (C.M.); phisit.s@cmu.ac.th (P.S.); jirarat4824@gmail.com (W.J.); 2 Program in Biotechnology, Faculty of Science, Maejo University, Chiang Mai 50290, Thailand; tpisithkul@gmail.com

**Keywords:** coffee cherry pulp, supercritical CO_2_ extraction, green valorization, untargeted metabolomics, DPP-IV inhibition, oligosaccharides, sustainable agriculture

## Abstract

Coffee cherry pulp (CCP), a major by-product and solid waste of coffee processing, is rich in phenolic compounds and pectin but remains underutilized. This study evaluated supercritical CO_2_ extraction with ethanol co-solvent (SFE-CO_2_) as a green alternative for recovering bioactive compounds from CCP and investigated the subsequent recovery of pectin-rich fractions and pectic oligosaccharides (POS). Using a one-factor-at-a-time (OFAT) approach, the selected SFE-CO_2_ conditions were 50% (*w*/*w*) ethanol, 55 °C, and 300 bar. Under these conditions, the SFE-CO_2_ extract exhibited total phenolic (4.26 ± 0.31 mg GAE/g extract) and total flavonoid contents (394.9 ± 10.5 μg QE/g extract) that were 1.6–5.4-fold higher than those of the hot-water and ethanol extracts. Antioxidant activities differed among extraction methods, with the SFE-CO_2_ extract showing higher activities in the DPPH and FRAP assays but lower ABTS radical-scavenging activity than the conventional extracts. In vitro DPP-IV inhibition (58.6–69.8% at 1.5 mg GAE/mL) did not differ significantly among the extracts. Untargeted UHPLC-MS/MS-based metabolomics (262 features) revealed distinct metabolite profiles among the extraction methods, with SFE-CO_2_ extracts enriched in chlorogenic acid, caffeoylquinic acid, and coumaroylquinic acid, indicating extraction-dependent selectivity. Hot-water pretreatment reduced the yield of the pectin-rich fraction obtained by microwave-assisted extraction to ≤8%, whereas ethanol- and SFE-CO_2_-treated residues yielded approximately 18–20%, comparable to untreated CCP. The SFE-CO_2_-treated residue yielded 10.88 ± 0.70% at 0.002% citric acid, comparable to the highest yield of untreated CCP at 0.2% citric acid (10.77 ± 0.34%). Enzymatic hydrolysis of the CCP-derived pectin-rich fractions released POSs with DP 1–4. Overall, the findings demonstrate the potential of an integrated green extraction strategy to enhance the utilization of CCP by combining selective bioactive compound recovery with downstream valorization of the residual biomass for the production of a pectin-rich fraction and pectic oligosaccharides, thereby contributing to the circular bioeconomy and sustainable utilization of coffee-processing by-products.

## 1. Introduction

Coffee is one of the most consumed beverages worldwide due to its distinctive flavor, aroma, and stimulating effects [1]. Coffee processing generates substantial quantities of by-products, including coffee cherry pulp (CCP), silver skin, and spent coffee grounds. CCP is the major by-product of coffee green bean processing, accounting for approximately 50% (*w*/*w*) of the fresh cherry weight [2]. As global coffee consumption continues to rise, CCP must be considered as a way of adding value, in alignment with the concepts of zero-waste management and the bio–circular–green (BCG) economy.

CCP contains a range of bioactive compounds, including phenolic acids, flavonoids, and other chemicals, that have been reported to exhibit antioxidant, anti-inflammatory, and enzyme-inhibitory activities [3]. As a result, various extraction technologies have been investigated for the recovery of these compounds from CCP, including conventional aqueous and ethanolic extraction as well as emerging green extraction technologies, such as high-pressure extraction and ultrasound-assisted extraction [4]. The extraction method used can substantially influence the extraction efficiency, the composition of the recovered compounds, and the resulting biological activities of the extracts [5,6]. Supercritical fluid extraction using CO_2_ (SFE-CO_2_), combined with ethanol as a co-solvent, has attracted interest because it enables the selective recovery of phenolic compounds and other moderately polar bioactive constituents while reducing the use of conventional organic solvents [7]. Recent work has demonstrated that ethanol-modified SFE-CO_2_ can produce extracts enriched in selected phenolic compounds and caffeine from coffee pulp, with extraction efficiency and selectivity influenced by the extraction conditions [8].

Although the bioactive properties of CCP extracts have been investigated using different extraction approaches, the relationship between extraction method, chemical composition, and biological activity remains insufficiently understood. In particular, the metabolites responsible for differences in bioactivity among CCP extracts obtained using different extraction methods have not been comprehensively characterized. Untargeted metabolomics using UHPLC-MS/MS represents a useful approach for profiling a broad range of metabolites without restricting the analysis to predefined compounds. This approach can therefore facilitate the identification of metabolites that discriminate among extraction treatments and may contribute to differences in biological activity [9]. In addition, although several biological activities have been reported for CCP extracts and their bioactive constituents [10], in vitro DPP-IV inhibitory activity of CCP extracts remains underexplored. To the best of our knowledge, this activity has not yet been systematically evaluated in CCP extracts obtained using different extraction methods. Therefore, DPP-IV inhibitory activity was included in the present study as an additional biological activity to explore the potential differences among CCP extracts rather than as the primary criterion for evaluating their bioactivity.

In addition to phenolic compounds, CCP contains structural polysaccharides, notably pectin, accounting for up to 15% of its dry weight [11]. The choice of extraction method may also affect the residual pectin content of the solid residue. Hot-water extraction can solubilize pectic polysaccharides from plant cell walls, whereas SFE-CO_2_ with ethanol as a co-solvent mainly extracts low- and intermediate-polarity compounds and is therefore expected to retain most pectic polysaccharides in the residue [12]. Pectin is conventionally recovered from such residues via acidified hot-water extraction, but this approach requires high acid concentrations and prolonged processing times [13]. Microwave-assisted extraction (MAE) offers a more sustainable alternative to conventional acid-hydrolysis pectin extraction, enabling rapid cell wall disruption with shorter processing times and, depending on operating conditions, reduced acid concentrations relative to conventional methods [14].

We hypothesized that the extraction approaches would differentially affect the recovery of bioactive compounds from CCP and the composition of the CCP residue, thereby influencing the subsequent recovery of the pectin-rich fraction. To address the knowledge gaps identified in the research conducted thus far, the present study aimed to investigate how the extraction approach influences the recovery and composition of bioactive compounds from CCP and the subsequent valorization of the residual biomass. SFE-CO_2_, ethanol, and hot-water extraction methods were compared in terms of bioactive compound recovery and antioxidant and in vitro DPP-IV inhibitory activities. Untargeted UHPLC-MS/MS-based metabolomics was further applied to characterize differences in the chemical profiles of the extracts and to identify metabolites associated with the observed bioactivities. In addition, the CCP residues were subjected to microwave-assisted extraction (MAE) for the subsequent recovery of a pectin-rich fraction, which was then assessed for its suitability for pectic oligosaccharide production. This integrated approach was proposed to provide a more comprehensive understanding of the relationship between the initial extraction process, extract composition and bioactivity, and the downstream valorization of CCP residues.

## 2. Materials and Methods

### 2.1. CCP Preparation and Chemicals

Coffee cherry pulp (CCP) was collected from the washed processing of Arabica coffee (*Coffea arabica*) at PANA Coffee Co., Ltd. (Chiang Mai, Thailand). Information regarding coffee cherry maturity and cultivation conditions was not available because these parameters were not systematically recorded at the source. Upon receipt, the CCP was dried in a hot-air oven at 60 °C for 48 h. The dried CCP was then milled using a coarse hammer mill (KN-200, Zhengzhou, China), packed in plastic bags, and stored under dark and dry conditions until further use. The particle size distribution of the CCP powder was determined by sieve analysis (Table S1). *Gly-Pro*-*p*-nitroanilide (G-0513-25MG) and Dipeptidyl peptidase-IV (DPP-IV, 317640-250MU) were purchased from Sigma-Aldrich (Saint Louis, MO, USA). The chemicals used for pectin extraction were food-grade reagents, while other chemicals were of analytical grade.

### 2.2. Extraction of Bioactive Compounds from CCP

Extraction conditions for bioactive compound recovery from coffee cherry pulp (CCP) by supercritical fluid extraction using CO_2_ (SFE-CO_2_) were selected using a one-factor-at-a-time (OFAT) approach. Three extraction factors were investigated, namely ethanol concentration as a co-solvent (0–50%, *w*/*w*), extraction temperature (35–75 °C), and extraction pressure (100–500 bar). Dried CCP (30 g) was loaded into a 50 mL extraction vessel and subjected to SFE-CO_2_ using a Spe-ed™ SFE 2 system (Applied Separations, Allentown, PA, USA) at the iExtract Pilot Plant, Food Innovation and Packaging Center, Chiang Mai University. Carbon dioxide was used as the primary extraction solvent at a flow rate of 3.0 L/min, while ethanol was employed as a co-solvent to enhance the extraction of polar bioactive compounds. The static and dynamic extraction times were 10 and 60 min, respectively. After the extraction, the collected extract was transferred to sealed containers. Ethanol was removed using a rotary vacuum evaporator (Büchi, Rotavapor^®^ R-3, Flawil, Switzerland) operated at 40 °C and 30 mbar for 2 h. The resulting extract was then stored in sealed containers at −20 °C in the dark until further analysis.

Hot-water and ethanol extraction were performed according to the method described previously [7], with some modifications. Hot-water extraction was performed by adding 10 g of CCP to 100 mL of distilled water. The mixture was shaken at 180 rpm for 3 h and incubated at 85 °C for 15 min. For ethanol extraction, 10 g of CCP was soaked in 100 mL of 55% (*v*/*v*) ethanol, then incubated at 25 °C in a shaking incubator (LSI-3016A, LabTech, Namyangju-si, Republic of Korea) with a shaking speed of 180 rpm for 24 h. The extracts were separated by filtration using filter paper. The remaining solid residue after bioactive compound extraction was collected and subsequently used for pectin extraction.

### 2.3. Recovery of Pectin-Rich Fraction from CCP Residue by Microwave-Assisted Extraction and Production of Pectic Oligosaccharides

Pectin was extracted from the obtained CCP residue by microwave extraction using a 23 L microwave (MS2322DW, LG, Bangkok, Thailand). The effects of citric acid concentrations (2−0.002% *w*/*v*, pH 2.25–4.19) and microwave power (90–600 W) on pectin yield were studied. To extract pectin from the CCP residue, 6 g of the residue was mixed with 60 mL of citric acid solution in a 250 mL Duran bottle. After extraction for 5 min, the mixture was filtered to separate the supernatant. Absolute ethanol (60 mL) was added to precipitate pectin. Pectin-rich fraction was separated by filtration through filter paper (Whatman no. 4) and dried at 60 °C in a hot air oven [15,16].

The pectin-rich fraction powder was then subjected to enzymatic hydrolysis to produce pectic oligosaccharides. The enzymatic reaction consisted of 1 g of the powder and 20 mL of 100 mM sodium citrate buffer, pH 4.5 (5% *w*/*v* substrate). The reaction was initiated by adding 0.2 mL of a commercial pectinase (Pectinex^®^ Ultra SP-L, Novonesis, Lyngby, Denmark), and the mixture was incubated at 35 °C in a water bath (NB20, Nüve, Ankara, Turkey) for 180 min. Commercial pectin from citrus peel (9000-69-5, Himedia, Maharashtra, India) was used as a positive control. Samples were periodically taken for analysis of reducing sugars using the DNS method [17]. In addition, the profile of pectic oligosaccharides was visualized by thin-layer chromatography (TLC) according to a previously described method with some modifications [18]. Samples were spotted on TLC Silica gel 60 F254 (Merck, Darmstadt, Germany) and separated by a mobile phase containing n-butanol, formic acid, and water in a ratio of 4:6:1. The TLC sheet was dried and sprayed with 0.2% (*w*/*v*) orcinol in 15% (*v*/*v*) of sulfuric acid in ethanol, and heated at 120 °C for 3 min. The POS standards, including mono-, di-, tri- and tetra-galacturonic acids, were purchased from Megazyme (Bray, Ireland).

### 2.4. Analytical Methods

#### 2.4.1. Determination of Total Phenolic Content

Total phenolic content (TPC) of the CCP extracts was determined using the previous method [19], with some modifications. Briefly, samples were dissolved in either deionized water or ethanol. The sample solution (80 μL) was then mixed with 320 μL of deionized water. To prepare the blank, deionized water was used in place of the sample. Subsequently, 100 μL of 0.2 M Folin–Ciocalteu reagent and 100 μL of 7% (*w*/*v*) sodium carbonate (Na_2_CO_3_) solution were added to the reaction mixture. The reaction mixture was then incubated in the dark at room temperature for 30 min to allow for color development. Absorbance was then measured at 765 nm against the blank using a spectrophotometer (GENESYS 40, Thermo Scientific, Madison, WI, USA). Gallic acid (Sigma-Aldrich, 398225) was used as the reference standard for calibration, and the results were expressed as mg gallic acid equivalent per g extract (mg GAE/g extract).

#### 2.4.2. Determination of Total Flavonoid Content

Total flavonoid content (TFC) was analyzed using the aluminum chloride colorimetric assay according to a previously reported method, with slight modifications [20]. Briefly, 250 μL of sample solution dissolved in either water or ethanol was mixed with 50 μL of 10% (*w*/*v*) aluminum chloride (AlCl_3_) solution. To prepare the blank, deionized water was used instead of the sample solution. Subsequently, 650 μL of absolute ethanol and 50 μL of 1 M sodium acetate buffer were added to the reaction mixture. The mixture was incubated at room temperature for 30 min in the dark before measuring absorbance at 415 nm against the blank. Quercetin (Sigma-Aldrich, Q4951) was used as the calibration standard, and the TFC values were expressed as μg quercetin equivalent per g extract (μg QE/g extract).

#### 2.4.3. Determination of Antioxidant Activity

The antioxidant activity of the CCP extracts was evaluated using ABTS, DPPH, and FRAP assays [21]. Trolox (Sigma-Aldrich, 238812) was used as the standard reference compound, and the results were expressed as µmol Trolox equivalent per g extract (µmol TE/g extract). The ABTS (Sigma-Aldrich, A1888) radical (ABTS^+^) working solution was prepared by reacting 7 mM ABTS with 2.45 mM potassium persulfate (K_2_S_2_O_8_) in the dark for 12–16 h and diluting with deionized water to an absorbance of 0.700 ± 0.020 at 734 nm. For the assay, samples were dissolved in either water or ethanol. The reaction mixture consisting of 50 μL of the sample and 850 μL of the ABTS^+^ solution was incubated in the dark for 30 min. The control was prepared using deionized water instead of the sample. Absorbance was measured at 734 nm. In addition, DPPH (Sigma-Aldrich, D9132) radical-scavenging activity was determined by mixing 500 μL of the sample solution with 500 μL of the 80 mg/L DPPH solution. The control consisted of DPPH solution mixed with deionized water instead of the sample. After incubation in the dark at room temperature for 30 min, the absorbance was measured at 517 nm. For the FRAP assay, FRAP reagent was freshly prepared by mixing 0.3 M acetate buffer (pH 3.6), 20 mM ferric chloride (FeCl_3_), and 10 mM TPTZ solution in 40 mM HCl at a ratio of 10:1:1 (*v*/*v*/*v*). The assay was performed by mixing 900 μL of FRAP reagent with 100 μL of sample solution, while deionized water was used for blank preparation. After incubation in the dark for 30 min, the absorbance was measured at 595 nm.

#### 2.4.4. DPP-IV Inhibitory Activity Assessment

Dipeptidyl peptidase-IV (DPP-IV) inhibitory activity was determined [22]. Prior to analysis, the TPC of CCP extracts obtained from SFE-CO_2_, ethanol, and hot-water extraction was adjusted to the same concentration of 1.5 mg GAE/mL to minimize the effect of phenolic concentration differences among samples. The substrate solution, 1.60 mM Gly-Pro-*p*-nitroanilide, and the enzyme solution containing 0.01 U/mL DPP-IV were freshly prepared using 100 mM Tris–HCl buffer (pH 8.0). For the assay, 25 μL of the diluted sample was mixed with 150 μL of DPP-IV solution and 50 μL of Tris–HCl buffer. The mixture was pre-incubated at 37 °C for 10 min to allow for interaction between the enzyme and sample compounds. Subsequently, 75 μL of 1.60 mM Gly-Pro-*p*-nitroanilide substrate solution was added to initiate the enzymatic reaction, followed by incubation at 37 °C for 60 min. After incubation, the reaction was terminated by adding 300 μL of absolute ethanol. Blank and control reactions were prepared according to the assay design to eliminate background interference from the enzyme, substrate, and sample matrix. The absorbance of the reaction mixtures was measured at 385 nm against the corresponding blank using a spectrophotometer. DPP-IV inhibitory activity was calculated and expressed as percentage inhibition. Sitagliptin (SML3205, Sigma-Aldrich) was used as a positive control (Figure S1).

### 2.5. UHPLC-MS/MS and Metabolomic Analysis

Each extraction method was represented by three independent extraction replicates, each of which was prepared and analyzed separately. For UHPLC-MS/MS analysis, a 20 µL aliquot of each extract was diluted tenfold with 180 µL of an appropriate solvent: 100% LC-MS-grade water for water-based extracts, 70% methanol for 50% and 55% ethanol extracts, and 100% methanol for absolute-ethanol extracts. Sulfadimethoxine was added as an internal standard at a final concentration of 25 ng/mL to monitor analytical performance; feature intensities were not normalized to the internal-standard response. Samples were centrifuged at 14,000 rpm for 10 min at room temperature, and the clarified supernatants were transferred to LC-MS vials. A 70% methanol extraction blank was included.

A pooled quality-control (QC) sample was prepared by combining equal 100 µL aliquots of the clarified supernatants from all study samples. Dilution-QC samples containing 0, 1, 10, 20, 50, and 80% pooled QC were also prepared. Sample injections were randomized within a dedicated analytical batch containing only samples and QCs from this study. Pooled-QC injections were interspersed throughout the sequence, generally after every two to three study samples, to monitor analytical consistency. All samples and QCs were prepared and analyzed within a single batch to minimize inter-batch variability; therefore, no inter-batch correction was applied.

Chromatographic separation was performed on an Agilent LC system equipped with a Poroshell 120 EC-C18 column (2.1 × 100 mm, 2.7 µm particle size) maintained at 50 °C. The mobile phases consisted of (A) 0.1% formic acid in water and (B) 0.1% formic acid in acetonitrile. The flow rate was 0.4 mL/min, and the injection volume was 10 µL. The gradient program was as follows: 0.0–0.5 min, 100% A; 0.5–10.5 min, linear gradient to 45% A/55% B; 10.5–12.5 min, linear gradient to 25% A/75% B; 12.5–14.0 min, linear gradient to 100% B; 14.0–17.0 min, held at 100% B; 17.0–17.5 min, returned to 100% A; and 17.5–20.0 min, re-equilibrated at 100% A.

Mass spectrometric analysis was performed using an Agilent 6545XT LC-QTOF-MS (Agilent Technologies, Inc., Santa Clara, CA, USA) operated in high-resolution mode. Data were acquired in separate positive- and negative-ion electrospray ionization (ESI) acquisitions, with each independently prepared extract injected once in each mode. Source parameters were as follows: drying-gas temperature, 325 °C; drying-gas flow, 13 L/min; sheath-gas temperature, 275 °C; sheath-gas flow, 12 L/min; nebulizer pressure, 45 psi; and capillary voltage, +4000 V in positive mode and −3000 V in negative mode. MS1 spectra were acquired over *m*/*z* 40–1700 and MS/MS spectra over *m*/*z* 25–1000 using data-dependent acquisition with a maximum of 10 precursors per cycle, a precursor-intensity threshold of 5000 counts, a retention-time threshold of 0.001%, and an isotope width of 1.3 *m*/*z*. Collision energies were 20 eV in positive mode and 10 eV in negative mode, and the acquisition rate was 3.35 spectra/s. Online mass-axis calibration used reference ions at *m*/*z* 121.0509 and 922.0098 in positive mode and *m*/*z* 112.9856 and 1033.9881 in negative mode. Only the negative-ESI dataset was used for feature annotation, PCA, PLS-DA, VIP selection, and heatmap construction; positive- and negative-mode data were not combined.

Authentic standards of quercetin (Sigma-Aldrich, Q4951), caffeic acid (TCI (Tokyo, Japan), C0002), chlorogenic acid (TCI, C0181), and gallic acid (Sigma-Aldrich, 398225) were analyzed in separate vials at 10, 50, and 100 µM within the same analytical sequence and under the same LC gradient and negative-ion MS conditions as the samples. A feature was considered standard-confirmed when its retention time, accurate precursor *m*/*z* (within 20 ppm), and MS/MS fragmentation pattern matched those of the authentic standard and the corresponding standard signal increased with concentration. Such assignments were classified as Metabolomics Standards Initiative (MSI) Level 1 identifications.

Raw Agilent data files (.d) were converted to .mzML format using MSConvert (ProteoWizard) and then to .mzXML format using the TOPPAS module in OpenMS. Negative-mode data were processed in MAVEN2 [23]. Mass slices were merged using an *m*/*z* tolerance of 20 ppm and a retention-time merge tolerance of 100 scans. Feature detection required a minimum signal-to-noise ratio of 3, a minimum sample-to-blank ratio of 3, a minimum highest-peak intensity of 50,000 ions, a minimum peak width of five scans, and valid peaks in at least two replicates per extraction group. MS/MS data were required for retained annotated features.

For features without authentic-standard confirmation, MS/MS spectra were matched in MAVEN2 against the MoNA-export-LC-MS-MS_Negative_Mode library using a compound *m*/*z* tolerance of 20 ppm and the Mzkitchen Metabolite Score. Library retention times were not used for these putative annotations. Candidate matches were manually reviewed using the number of matched fragment ions (fragNumIonsMatched) and MS2 spectral-purity values. Features lacking MS/MS spectra, showing insufficient fragment support, or with poor precursor-isolation purity were excluded. Because matched-fragment number and MS2 purity were evaluated jointly during spectral review, no single numerical cutoff was applied. Library-supported assignments without authentic-standard confirmation were reported as MSI Level 2 putative annotations when the evidence supported a specific compound. Assignments that could not distinguish a unique structure were reported at the compound-class level (MSI Level 3) or as unknown features (MSI Level 4). Feature IDs, retention times, observed *m*/*z* values, proposed ions/adducts, MS/MS evidence, authentic-standard use, and MSI confidence levels are provided in Table S2.

The filtered negative-ESI feature-intensity matrix was analyzed in MetaboAnalyst 6.0. No sample-wise intensity normalization was applied before import. For PCA and PLS-DA, the data were mean-centered and Pareto-scaled. PLS-DA models containing up to three components were evaluated by leave-one-out cross-validation (LOOCV), with Q^2^ used to select the optimal model; model performance was summarized using accuracy, R^2^, and Q^2^. The statistical significance of class discrimination was assessed using 1000 permutations of the class labels, with prediction accuracy as the test statistic. Features with variable-importance-in-projection scores ≥ 1.0 were selected for heatmap visualization. For the heatmap, intensities were transformed to feature-wise z-scores across samples, and hierarchical clustering was performed using uncentered-correlation distance and complete linkage. Thus, the heatmap represents relative intensity patterns within each feature across samples, rather than quantitative abundance comparisons among different features.

### 2.6. Statistical Analysis

The experiments were carried out in triplicate (*n* = 3). The results are represented as the means of independent biological replicates ± standard deviation (SD). Statistical analysis was performed using Tukey’s HSD (*p* < 0.05) on SPSS Statistics for Windows v.17.0 (SPSS Inc., Chicago, IL, USA).

## 3. Results and Discussion

### 3.1. Effects of Ethanol Concentration, Temperature, and Pressure on the Extraction of Bioactive Compounds from CCP Using SFE-CO_2_

**Figure 1:**
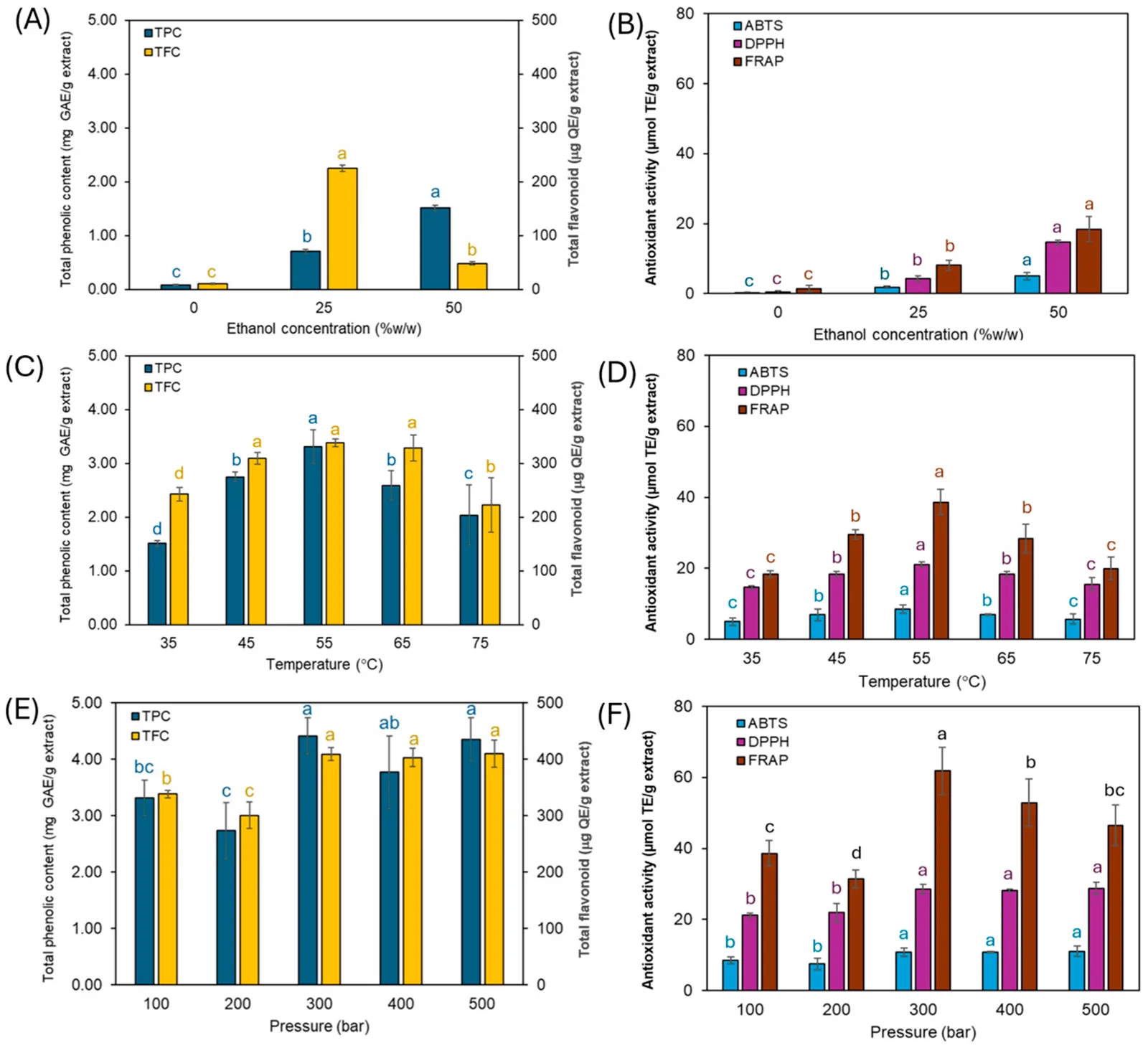
Effects of three independent factors on the extraction of bioactive compounds via SFE-CO_2_ based on the one-factor-at-a-time (OFAT) approach. Shown are the effects of (**A**,**B**) ethanol concentration; (**C**,**D**) extraction temperature; and (**E**,**F**) extraction pressure on total phenolic content (TPC), total flavonoid content (TFC), and antioxidant activity of CCP extracts. Antioxidant activity was evaluated using ABTS, DPPH, and FRAP assays. Different letters (a–d) indicate significant differences among extraction conditions according to Tukey’s HSD (*p* < 0.05).

To select the conditions for extracting bioactive compounds from CCP using SFE-CO_2_, the effects of ethanol concentration, extraction temperature, and extraction pressure on TPC, TFC, and antioxidant activity were investigated. Initially, the effect of co-solvent concentration was evaluated by varying ethanol concentration from 0 to 50% (*w*/*w*). As shown in Figure 1A, the addition of ethanol significantly improved the extraction of phenolic and flavonoid compounds from CCP due to the increased polarity of the supercritical solvent system, which enhanced the solubility of polar compounds [24,25]. The highest TPC value of 1.51 ± 0.05 mg GAE/g extract was obtained at 50% (*w*/*w*) ethanol. Similarly, the most significant antioxidant activities determined by ABTS, DPPH, and FRAP assays were also observed at 50% ethanol, with values of 5.00 ± 0.18, 14.60 ± 0.52, and 18.40 ± 0.96 µmol TE/g extract, respectively (Figure 1B). A previous study also applied 9% ethanol as a co-solvent for extracting bioactive compounds from coffee pulp using SFE-CO_2_ [8]. Based on these results, 50% (*w*/*w*) ethanol was selected for further study.

The effect of extraction temperature was subsequently investigated at 35–75 °C while maintaining the ethanol concentration at 50% (*w*/*w*). Increasing the temperature from 35 to 55 °C enhanced TPC, TFC, and antioxidant activity. However, extraction at temperatures above 55 °C resulted in reduced antioxidant activity (Figure 1C), which may be associated with the thermal degradation of heat-sensitive compounds [7]. The highest TPC and TFC values, corresponding to 3.31 ± 0.31 mg GAE/g extract and 268.15 ± 5.34 μg QE/g extract, respectively, were achieved at 55 °C. The highest antioxidant activities measured by ABTS, DPPH, and FRAP assays were also obtained at this temperature, with values of 8.52 ± 1.02, 26.65 ± 0.89, and 38.63 ± 3.59 µmol TE/g extract, respectively (Figure 1D). Consequently, 55 °C was selected for the subsequent experiments.

Extraction pressure was then evaluated in the range of 100–500 bar using 50% (*w*/*w*) ethanol at 55 °C. Among the tested conditions, extraction at 300 bar resulted in the highest recovery of bioactive compounds and antioxidant activity. The extract obtained at 300 bar exhibited TPC and TFC values of 4.26 ± 0.31 mg GAE/g extract and 394.9 ± 10.5 μg QE/g extract, respectively (Figure 1E). In addition, the highest antioxidant activities determined by ABTS, DPPH, and FRAP assays were 10.80 ± 1.17, 36.05 ± 1.68, and 61.82 ± 6.67 µmol TE/g extract, respectively (Figure 1F). Therefore, the selected SFE-CO_2_ extraction conditions for recovering bioactive compounds from CCP were determined to be 50% (*w*/*w*) ethanol as a co-solvent, an extraction temperature of 55 °C, and an extraction pressure of 300 bar. Under these conditions, the highest TPC, TFC, and antioxidant activities among the tested conditions were obtained.

### 3.2. Effects of Extraction Methods on Recovery of Bioactive Compounds, Antioxidant Activity, and DPP-IV Inhibitory Activity from CCP

To compare the extraction efficiency of SFE-CO_2_ with that of conventional extraction methods, CCP was also extracted via hot-water and ethanol extraction (Table 1). The results demonstrated that SFE-CO_2_ extraction resulted in significantly higher TPC and TFC values than both hot-water and ethanol extraction. Similarly, the antioxidant activities determined by the DPPH and FRAP assays were enhanced in the SFE-CO_2_ extract. In contrast, ABTS radical-scavenging activity was lowest in the SFE-CO_2_ extract among the extraction methods evaluated. These differences may be explained by the distinct reaction mechanisms of the antioxidant assays. The DPPH assay primarily measures the ability of antioxidants to donate hydrogen atoms or electrons to a lipophilic radical, whereas the FRAP assay evaluates the reducing power of antioxidants through electron transfer. In contrast, the ABTS assay can detect both hydrophilic and lipophilic antioxidant compounds and is often influenced by a broader range of antioxidant constituents. Therefore, the higher DPPH and FRAP activities observed in the SFE-CO_2_ extract suggest an enrichment of compounds with strong electron-donating and reducing capacities, while the lower ABTS activity indicates a different antioxidant response profile depending on the assays [6,26,27].

In contrast to antioxidant activity, DPP-IV inhibitory activity did not significantly differ among extraction methods. DPP-IV is an important enzyme involved in glucose metabolism through the regulation of incretin hormones associated with insulin and glucagon secretion. Therefore, inhibition of DPP-IV has been recognized as a therapeutic and preventive strategy for type 2 diabetes mellitus [22,28]. The observed inhibitory activity of CCP extracts suggests the potential application of CCP-derived bioactive compounds as functional ingredients for improved metabolic health.

The differences between antioxidant activity and DPP-IV inhibition among extraction methods may indicate that compounds responsible for antioxidant activity are not solely associated with DPP-IV inhibitory effects. Therefore, further identification of bioactive compounds by UHPLC-MS/MS analysis is necessary to determine the major compounds contributing to these biological activities. In this study, we also investigated the solid residue remaining after extraction for pectin recovery to maximize the utilization and value of CCP as a sustainable agro-industrial by-product.

The extraction yield differed significantly among the extraction methods. Hot-water extraction produced the highest extraction yield, followed by ethanol extraction, whereas SFE-CO_2_ extraction resulted in the lowest yield. Despite its lower overall extraction yield, SFE-CO_2_ extraction resulted in higher TPC and TFC values than the conventional extraction methods. This finding suggests that SFE-CO_2_ may provide a more selective extraction of phenolic and flavonoid compounds, whereas hot-water extraction may co-extract a broader range of water-soluble components, including sugars and polysaccharides.

### 3.3. UHPLC-MS/MS Profiling of CCP Extracts from Different Extraction Methods

**Figure 2:**
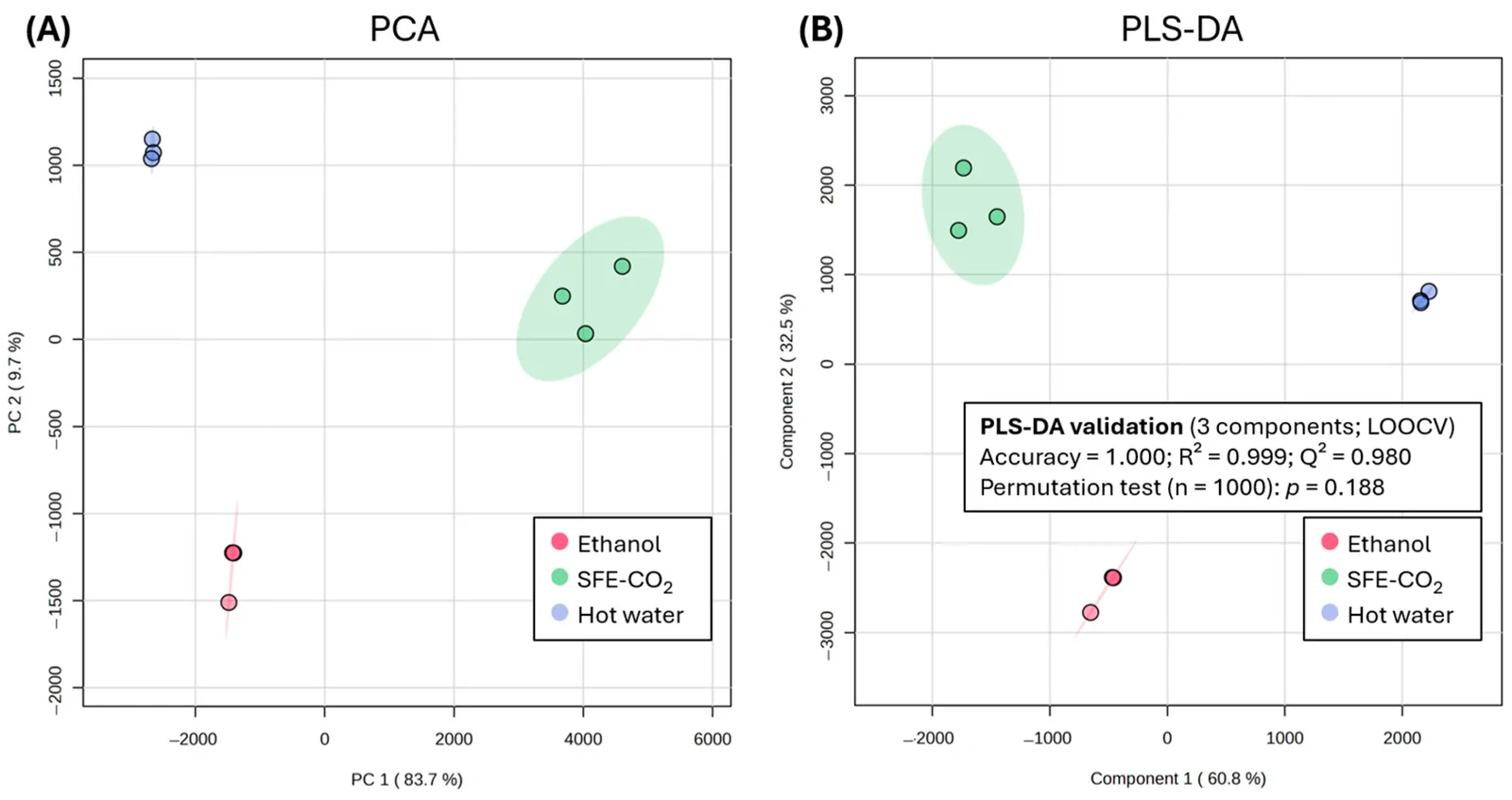
Overview of metabolite profiles of CCP extracts obtained using different extraction methods. (**A**) PCA 2D score plot showing the separation between extracts; (**B**) PLS-DA 2D score plot further highlighting the clear clustering between groups.

To further investigate the influence of extraction methods on the metabolite profile of CCP extracts, untargeted metabolomics analysis in negative electrospray ionization (ESI) mode was performed on extracts obtained from hot-water, ethanol, and SFE-CO_2_ extraction. A total of 262 metabolite features were detected within the chemical classes, including phenolic acids, flavonoids, organic acids, indole derivatives, coumarins, fatty acids, and nucleoside-related compounds (Table S2). Principal component analysis (PCA) was then performed (Figure 2A). The PCA score plot revealed clear separation among all three extraction groups, with PC1 and PC2 values of 83.7 and 9.7%, respectively. SFE-CO_2_ extracts formed a distinct cluster, which separated from both hot-water and ethanol extracts, suggesting that supercritical extraction generated a unique metabolite profile.

**Figure 3:**
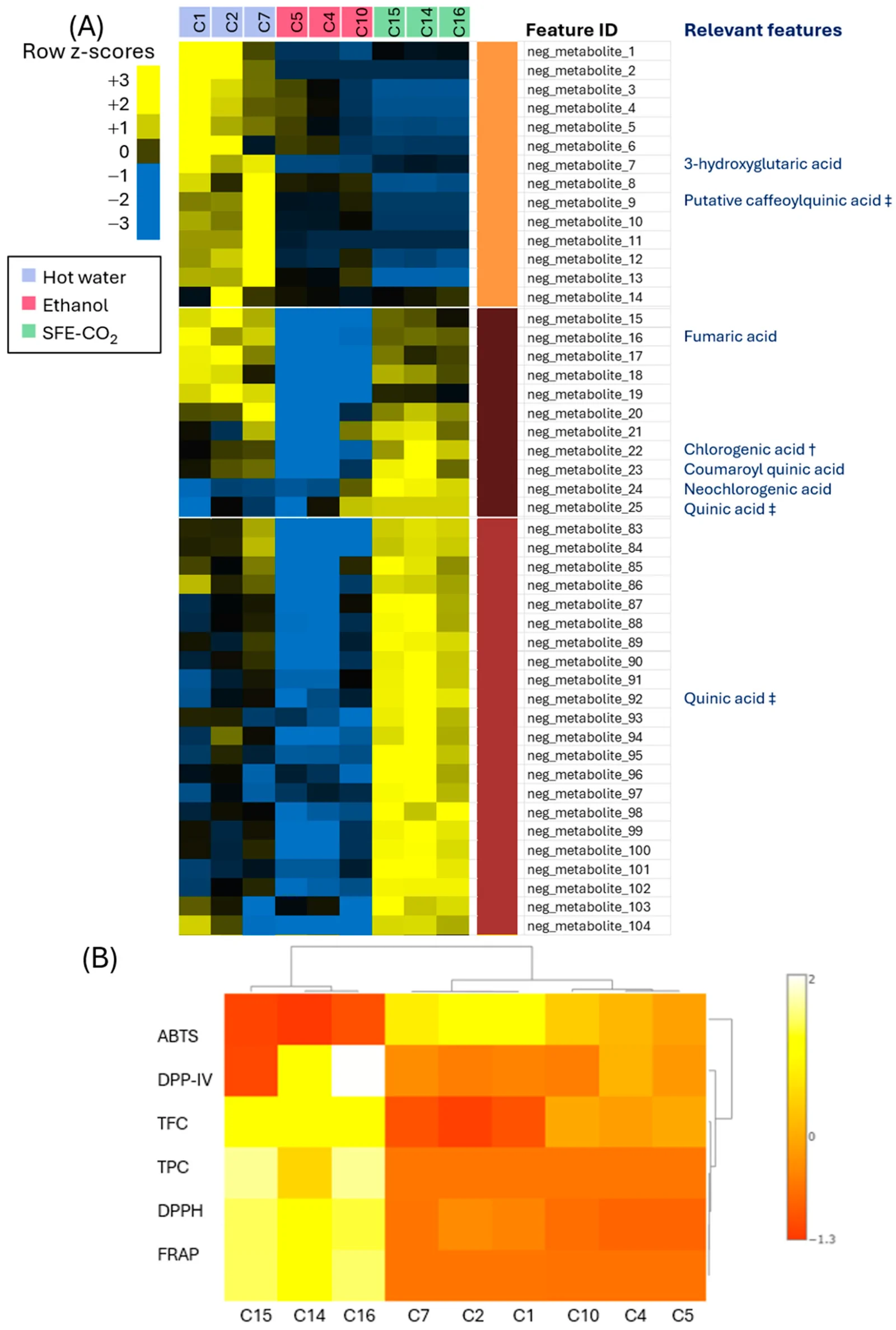
(**A**) Heat map of negative-ionization-mode LC–MS features across three independent extractions for different extraction methods: hot-water extraction (C1, C2 and C7), ethanol extraction (C4, C5, C10) and SFE-CO_2_ extraction (C14, C15 and C16). Feature intensities are presented as row-wise z-scores, with yellow and blue indicating relatively higher and lower intensities, respectively. Rows were grouped using Cluster3.0 (http://bonsai.hgc.jp/~mdehoon/software/cluster/ (accessed on 31 August 2026)) with uncentered-correlation distance. Only selected features discussed in the text are labeled; complete feature IDs, annotations, and identification-confidence levels are provided in Table S2. † Authentic-standard-confirmed identification; ‡ putative annotation based on MS/MS library matching. (**B**) Correlation analysis of metabolite intensity and TPC, TFC and biological activity by MetaboAnalyst (https://www.metaboanalyst.ca/ (accessed 31 on August 2026)).

In addition to unsupervised PCA, supervised partial least squares discriminant analysis (PLS-DA) also revealed clear clustering of metabolite profiles according to treatment groups, indicating the influence of extraction methods on the metabolite profiles of each sample (Figure 2B). Metabolites with variable importance in projection (VIP) scores were identified as significant discriminant features. These discriminant metabolites were subsequently used to construct a heatmap to visualize their extraction-dependent distribution patterns (Figure 3A). The heatmap revealed distinct distribution profiles among the key discriminant metabolites. Phenolic acids and flavonoids, including chlorogenic acid, caffeoylquinic acid, and coumaroylquinic acid, were detected in all three extraction methods but consistently showed the highest relative intensities in the SFE-CO_2_ extracts. These findings were consistent with the significantly higher TPC and TFC values observed in the SFE-CO_2_ extract (Table 1).

The enrichment of chlorogenic acid and related hydroxycinnamic acid derivatives in the SFE-CO_2_ extract might be consistent with its higher DPPH and FRAP activities, as these compounds have been widely reported to exhibit antioxidant activity [27,28,29]. Computational modeling has further predicted hydroperoxyl radical-scavenging and xanthine oxidase-inhibitory activity for related caffeoylquinic acid derivatives [30]. However, the contribution of individual metabolites to the antioxidant activities observed in the present study was not experimentally confirmed.

In addition, chlorogenic acid has been computationally predicted via in silico molecular dynamics simulations to inhibit DPP-IV activity through interactions with the hydrophobic S1 and S2 subsites of the enzyme’s active site [30,31]. However, to the best of our knowledge, evidence regarding the DPP-IV inhibitory activity of caffeoylquinic acid and coumaroylquinic acid derivatives remains limited. Accordingly, a direct relationship between the abundance of chlorogenic acid or other phenolic compounds and the observed in vitro DPP-IV inhibitory activity will be of interest in our future research.

In contrast, organic acids and indole derivatives were predominantly recovered in the hot-water fraction, especially fumaric acid. The high contribution of these polar metabolites in the aqueous extract is consistent with their hydrophilic characteristics, and fumaric acid has previously been reported among the organic acids present in coffee pulp [32]. Notably, certain organic acids co-extracted in the hot-water fraction, such as citric acid, have also been reported to exhibit metal-chelating antioxidant activity [33], although this property has not been specifically confirmed for fumaric acid in the present study.

Further analysis revealed positive correlations between the relative intensities of several discriminant metabolites and TPC, TFC, and antioxidant activities (according to DPPH and FRAP assays) (Figure 3B). In contrast, the metabolite profile of the hot-water extract was more closely associated with ABTS radical-scavenging activity. These correlations suggest potential relationships between extraction-dependent metabolite profiles and the observed bioactivities. Taken together, the multivariate analysis demonstrated that extraction methods significantly and selectively influenced the recovery of bioactive compounds from CCP. SFE-CO_2_ extraction provided the broadest recovery of phenolic acids and flavonoids likely associated with antioxidant activity. Hot-water extraction selectively recovered polar organic acids and indole derivatives, while ethanol extraction was enriched in intermediate-polarity antioxidants and flavonoid glycosides.

### 3.4. Effect of Extraction Pretreatment on Pectin Recovery from CCP Residue by Microwave-Assisted Extraction

**Figure 4:**
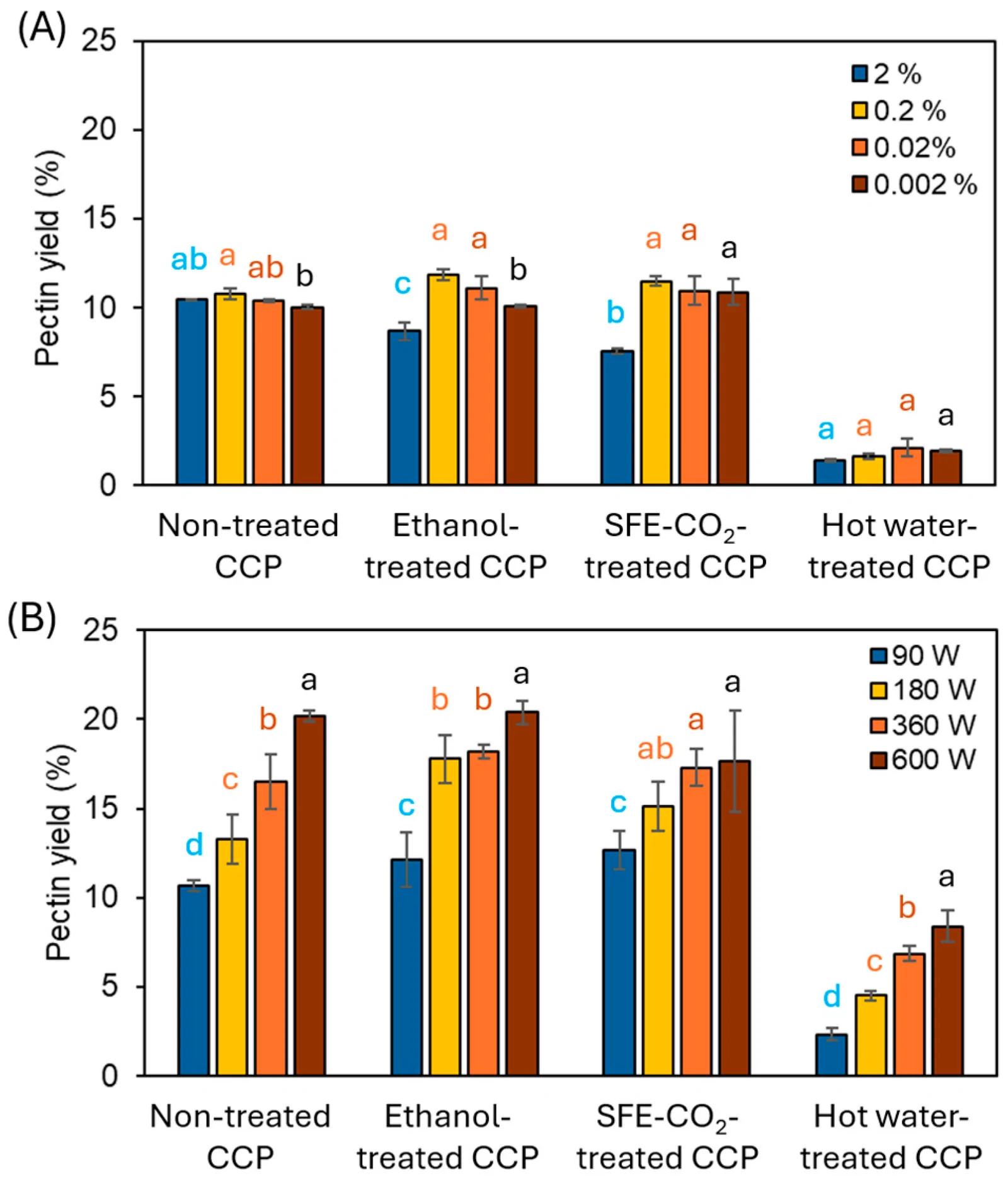
Effect of extraction pretreatment on pectin recovery from CCP residue by microwave-assisted extraction. (**A**) Effect of citric acid concentration and (**B**) microwave power on pectin recovery from different CCP residues. The yield is expressed as g pectin-rich fraction per 100 g CCP residue used for the extraction (%). Different letters (a–d) indicate significant differences among extraction conditions within the same substrate, according to Tukey’s HSD test (*p* < 0.05).

The CCP residue obtained after hot-water extraction (hot-water-treated CCP) exhibited the lowest pectin yield (≤8%). In contrast, higher pectin recovery was observed in the ethanol-treated and SFE-CO_2_-treated CCP residues, with values > 15%. Notably, the pectin recovery from the ethanol-treated and SFE-CO_2_-treated CCP residues was comparable to that from non-treated CCP. These findings suggest that pectin loss may occur during the initial hot-water extraction step, rather than being a disadvantage of the subsequent microwave-assisted extraction (MAE) process. In addition, the comparable pectin recovery observed after ethanol and SFE-CO_2_ pretreatments suggests that these non-aqueous or low-polarity extraction processes do not substantially remove the polysaccharide fraction of CCP. Notably, SFE-CO_2_ pretreatment reduced the citric acid concentration required to achieve the highest pectin yield among the tested conditions. Non-pretreated CCP required 0.2% citric acid to achieve its highest pectin yield (10.77 ± 0.34%). In comparison, the SFE-CO_2_-treated residue achieved a comparable yield (10.88 ± 0.70%) at a citric acid concentration of 0.002% (Figure 4A and Figure S2). These results might be explained by the removal of lipophilic surface constituents by SFE-CO_2_ (Table S2). In addition to phenolic compounds, SFE-CO_2_, alongside ethanol as a co-solvent, could extract lipophilic compounds such as lipids and tocopherols from coffee pulp [8]. The removal of such compounds, including cuticular waxes, may reduce the hydrophobic barrier that limits the penetration of the dilute aqueous acid into the cell wall matrix during MAE. A previous study showed that supercritical CO_2_ (scCO_2_) extraction of waxes from Miscanthus (silver grass) at 350 bar and 50 °C increased sugar release during subsequent enzymatic saccharification by approximately 20%, whereas the alcohol-insoluble residue did not show any improvement. In addition, scanning electron microscopy (SEM) also revealed rougher surfaces after scCO_2_ treatment [34]. Similarly, in the present study, the ethanol-treated residue showed a significant decrease in yield at 0.002% citric acid, whereas the SFE-CO_2_-treated residue did not.

The effect of microwave power on pectin yield was subsequently investigated at the selected citric acid concentration. As shown in Figure 4B, pectin yields increased with microwave power for all residues. Microwave power is a crucial factor affecting the pectin extraction efficiency of MAE [35]. The resulting pectin-rich fraction was subsequently subjected to enzymatic hydrolysis using a commercial pectinase to evaluate its potential as a substrate for oligosaccharide production.

### 3.5. Production of Pectic Oligosaccharides from Pectin-Rich Fraction

To evaluate the suitability of the recovered pectin-rich fractions as substrates for pectic oligosaccharide (POS) production, enzymatic hydrolysis was performed using a commercial pectinase. An increase in reducing sugar content was observed for all pectin-rich fractions and commercial citrus pectin during hydrolysis (Table 2). For all substrates, reducing sugar release increased rapidly within the first 15 min, followed by a gradual decrease in the hydrolysis rate, reaching a plateau after approximately 120 min.

Among the CCP-derived substrates, the pectin-rich fraction recovered from hot-water-treated CCP residue produced the highest amount of reducing sugars, which was significantly higher *(p* < 0.05) than those obtained from non-treated, SFE-CO_2_-treated, and ethanol-treated CCP residues. However, the amount of reducing sugars released from all CCP-derived pectin-rich fractions was lower than that obtained from commercial citrus pectin. This difference may reflect variations in the composition and physicochemical properties of the recovered pectin-rich fractions relative to commercial pectin. However, these characteristics were not determined in the present study and therefore needed further investigation.

**Figure 5:**
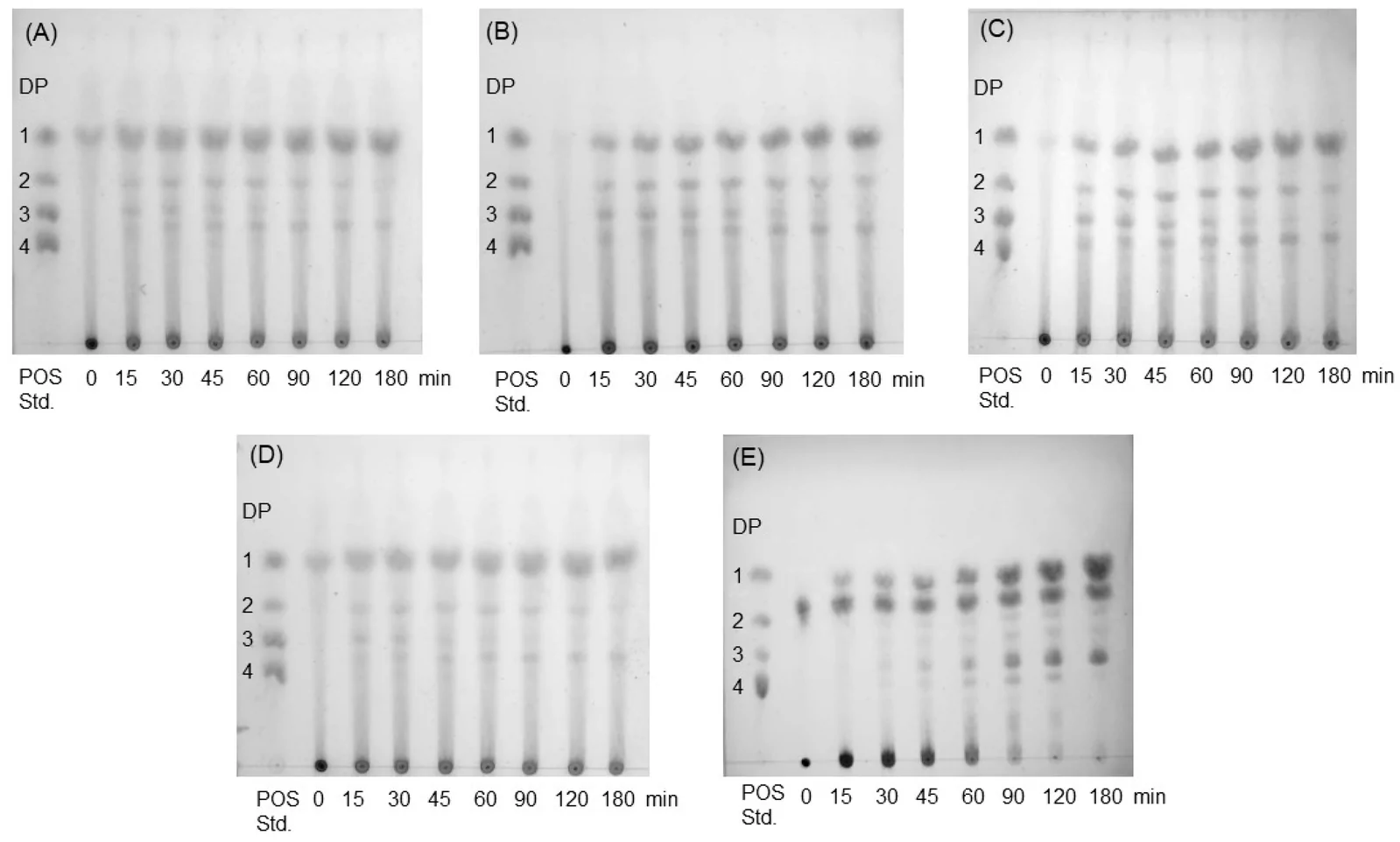
TLC chromatogram of pectic oligosaccharides (POS) produced from 5% (*w*/*v*) pectin-rich fractions recovered from non-treated CCP (**A**), SFE-CO_2_-treated CCP residue (**B**), hot-water-treated CCP residue (**C**), and ethanol-treated CCP residue (**D**), compared with commercial citrus pectin (**E**). Hydrolysis was carried out in 100 mM sodium citrate buffer (pH 4.5) using Pectinex^®^ Ultra SP-L (Novonesis; 0.2 mL per reaction) at 35 °C for 180 min. Galacturonic acid (DP 1), di-galacturonic acid (DP2), tri-galacturonic acid (DP 3), and tetra-galacturonic acid (DP 4) were included as reference standards.

To further examine the hydrolysis products, samples were analyzed by thin-layer chromatography (TLC). As shown in Figure 5A–D, enzymatic hydrolysis of all CCP-derived pectin-rich fractions generated galacturonic acid (GalA), together with di-, tri-, and tetra-galacturonic acid oligomers. Notably, the TLC profiles obtained from the non-treated CCP and SFE-CO_2_-treated CCP residues were similar, suggesting that the hydrolysis products generated from these two substrates were comparable under the conditions tested. These results indicate that pectin-rich fractions recovered from CCP can serve as substrates for POS production, although differences in hydrolysis efficiency were observed among the pretreatment conditions.

## 4. Conclusions

This study demonstrated that SFE-CO_2_ combined with ethanol as a co-solvent is an effective green method for recovering bioactive compounds from coffee cherry pulp (CCP). The investigated CCP extracts displayed both antioxidant activity and DPP-IV inhibitory activity, and we observed differences in TPC, TFC, and antioxidant activities among extraction methods. The SFE-CO_2_ extract showed higher activities in the DPPH and FRAP assays but the lowest ABTS radical-scavenging activity, whereas all extracts exhibited comparable in vitro DPP-IV inhibition. UHPLC-MS/MS-based metabolomics further revealed that the extraction method selectively shaped the metabolite profile of CCP extracts and the correlation of those metabolites to bioactivity. In addition, the effect of the initial extraction method on subsequent pectin recovery was also determined. SFE-CO_2_ extraction was able to preserve the pectin in the treated CCP residue and required a lower citric acid concentration to achieve comparable pectin yields via microwave-assisted extraction. Moreover, the obtained pectin-rich fraction proved to be a promising substrate for producing POS. Taken together, these findings support an integrated valorization strategy for CCP to produce bioactive compounds, pectin-rich fractions and POS. Future work should focus on assessing the feasibility of this integrated approach, isolation and validation of the specific metabolites responsible for in vitro DPP-IV inhibitory activity, the cytotoxicity of these metabolites, and evaluation of the prebiotic properties of POS.

## Figures and Tables

**Table 1:** Comparison of different techniques for extracting bioactive compounds from coffee cherry pulp (CCP).

	Hot Water ^1^	Ethanol ^1^	SFE-CO_2_ ^1^
TPC (mg GAE/g extract)	2.59 ± 0.38 ^b^	2.01 ± 0.24 ^c^	4.26 ± 0.31 ^a^
TFC (μg QE/g extract)	73.0 ± 11.7 ^c^	188.4 ± 34.3 ^b^	394.9 ± 10.5 ^a^
ABTS (μmol TE/g extract)	29.31 ± 7.12 ^a^	18.74 ± 2.27 ^b^	10.80 ± 1.17 ^c^
DPPH (μmol TE/g extract)	14.39 ± 1.11 ^b^	9.98 ± 1.19 ^c^	36.05 ± 1.68 ^a^
FRAP (μmol TE/g extract)	4.91 ± 0.81 ^b^	3.98 ± 0.68 ^b^	61.82 ± 6.67 ^a^
DPP-IV inhibition (%)	62.42 ± 13.40 ^a^	58.58 ± 4.75 ^a^	69.75 ± 23.46 ^a^
Yield (g extract/100 g dry CCP)	3.26 ± 0.04 ^a^	2.41 ± 0.10 ^b^	0.80 ± 0.01 ^c^

^1^ The experiment was performed in triplicate (*n* = 3), and the results are represented as the means of independent biological replicates ± standard deviation (SD). Different letters (a–c) within the same row indicate significant differences among treatments, as determined by Tukey’s HSD test (*p* < 0.05).

**Table 2:** Reducing sugar contents released during enzymatic hydrolysis of pectin-rich fractions for producing POS.

Time (min)	Reducing Sugar (mg/g Substrate)				
	Non-Treated CCP Residue	SFE-CO_2_-Treated CCP Residue	Ethanol-Treated CCP Residue	Hot-Water-Treated CCP Residue	Commercial Citrus Pectin
0	n.d.	n.d.	n.d.	n.d.	n.d.
15	151.9 ± 61.0 ^c^	140.5 ± 21.7 ^b^	151.9 ± 63.7 ^b^	237.8 ± 44.2 ^c^	134.5 ± 19.2 ^f^
30	188.2 ± 1.4 ^b^	168.9 ± 1.6 ^b^	186.3 ± 14.8 ^b^	335.6 ± 32.5 ^b^	194.9 ± 16.8 ^e^
45	200.7 ± 5.1 ^b^	195.5 ± 18.6 ^ab^	252.5 ± 34.6 ^a^	330.1 ± 30.1 ^ab^	265.7 ± 13.2 ^de^
60	207.3 ± 7.6 ^b^	198.2 ± 3.0 ^ab^	279.7 ± 11.1 ^a^	347.5 ± 0.2 ^ab^	303.8 ± 1.0 ^d^
90	218.0 ± 17.0 ^b^	241.4 ± 56.0 ^a^	284.3 ± 48.7 ^a^	366.0 ± 0.7 ^ab^	370.0 ± 47.7 ^c^
120	271.4 ± 25.7 ^a^	240.8 ± 20.3 ^a^	281.1 ± 56.1 ^a^	373.1 ± 14.3 ^ab^	426.4 ± 32.7 ^b^
180	253.3 ± 4.5 ^a^	260.3 ± 37.2 ^a^	309.2 ± 8.5 ^a^	380.7 ± 30.0 ^a^	479.8 ± 12.8 ^a^

The experiment was performed in triplicate (*n* = 3), the values were subtracted by the initial reducing sugar concentration at 0 min, and the results are represented as the means of independent biological replicates ± standard deviation (SD). Different letters (a–f) within the same column indicate significant differences among hydrolysis times, as determined by Tukey’s HSD test (*p* < 0.05). n.d. means not detected.

## Data Availability

The original contributions presented in the study are included in the article, further inquiries can be directed to the corresponding author.

## Supplementary Materials

The following supporting information can be downloaded at: https://www.mdpi.com/article/10.3390/foods15193555/s1. Table S1. The particle size distribution of the CCP powder used in this study. Table S2. Metabolomic data. Figure S1. Determination of the IC_50_ value of sitagliptin (SML3205, Sigma-Aldrich) as a positive control for the DPP-IV inhibition assay. Figure S2. Photographs of pectin-rich fractions recovered from (A) non-treated CCP, (B) SFE-CO_2_-treated CCP, (C) ethanol-treated CCP residue, and (D) hot water-treated CCP residue using 2% (*w*/*v*) citric acid.

## References

[1] Pothimoi S., Seesuriyachan P., Chaiyaso T., Insomphun C., Yakul K., Phimolsiripol Y., Moukamnerd C. (2026). Enhanced Sensory, Antioxidant, and Non-Toxic Anti-Diabetes Beverage Development via Co-Culture Fermentation of *Lactiplantibacillus plantarum* and *Saccharomyces boulardii* in Coffee Cherry Pulp Extracts. Food Chem. X.

[2] Heeger A., Kosińska-Cagnazzo A., Cantergiani E., Andlauer W. (2017). Bioactives of Coffee Cherry Pulp and Its Utilisation for Production of Cascara Beverage. Food Chem..

[3] Hu S., Gil-Ramírez A., Martín-Trueba M., Benítez V., Aguilera Y., Martín-Cabrejas M.A. (2023). Valorization of Coffee Pulp as Bioactive Food Ingredient by Sustainable Extraction Methodologies. Curr. Res. Food Sci..

[4] Vera W., Quevedo-Olaya J.L., Samaniego-Rafaele C., Culqui-Arce C., Sánchez-Chero M.J., Quispe-Santivañez G.W., Salvador-Reyes R. (2026). Emerging Technologies for Sustainable Extraction and Valorization of Bioactive Compounds from Coffee Beans and By-Products: Principles, Bioactivity Enhancement, and Industrial Perspectives. Biomass.

[5] Prado J.P.Z., Basso R.C., Rodrigues C.E.d.C. (2025). Extraction of Biomolecules from Coffee and Cocoa Agroindustry Byproducts Using Alternative Solvents. Foods.

[6] Kusumocahyo S.P., Wijaya S., Dewi A.A.C., Rahmawati D., Widiputri D.I. (2020). Optimization of the Extraction Process of Coffee Pulp as a Source of Antioxidant. IOP Conf. Ser. Earth Environ. Sci..

[7] Thai L.Q., Niwat C., Qin S., Konsue N. (2024). Supercritical Carbon Dioxide and Ethanol-Assisted Extraction of Bioactive Compounds from Bourbon, Catimor, and Caturra Coffee Pulp for Maximized Antioxidant and Therapeutic Properties. Future Foods.

[8] Hu S., Martín-Cabrejas M.Á., Hernández D.M., Martín-Trueba M., Cañas S., Rebollo-Hernanz M., Aguilera Y., Benítez V., Gil-Ramírez A. (2025). Tailored Recovery of Antioxidant Fractions Enriched in Caffeine and Phenolic Compounds from Coffee Pulp Using Ethanol-Modified Supercritical Carbon Dioxide. Food Res. Int..

[9] Thacharoen C., Inkaewwong T., Jumpathong W., Kaewsapsak P., Rattanapot T., Pisithkul T. (2025). Metabolomic Profiling and Anti-*Helicobacter pylori* Activity of *Caulerpa lentillifera* (Sea Grape) Extract. Mar. Drugs.

[10] Yakul K., Insomphun C., Seesuriyachan P., Chaiyaso T., Htike S.L., Phimolsiripol Y., Castagnini J.M., Moukamnerd C. (2026). Controlled Fermentation and Integrated Valorization of Coffee Cherry Pulp: Applications in Food, Bioactive, Biopolymers, and Animal Feed. Fermentation.

[11] Dao D.N., Le P.H., Do D.X., Dang T.M.Q., Nguyen S.K., Nguyen V. (2023). Pectin and Cellulose Extracted from Coffee Pulps and Their Potential in Formulating Biopolymer Films. Biomass Convers. Biorefin..

[12] Reichembach L.H., Kaminski G.K., Maurer J.B.B., de Oliveira Petkowicz C.L. (2024). Fractionation and Characterization of Cell Wall Polysaccharides from Coffee (*Coffea Arabica* L.) Pulp. Carbohydr. Polym..

[13] Biratu G., Woldemariam H.W., Gonfa G. (2024). Optimization of Pectin Yield Extracted from Coffee Arabica Pulp Using Response Surface Methodology. Heliyon.

[14] Sarah M., Hasibuan I.M., Misran E., Maulina S. (2022). Optimization of Microwave-Assisted Pectin Extraction from Cocoa Pod Husk. Molecules.

[15] Wongkaew M., Sommano S.R., Tangpao T., Rachtanapun P., Jantanasakulwong K. (2020). Mango Peel Pectin by Microwave-Assisted Extraction and Its Use as Fat Replacement in Dried Chinese Sausage. Foods.

[16] Liang Y., Yang Y., Zheng L., Zheng X., Xiao D., Wang S., Ai B., Sheng Z. (2022). Extraction of Pectin from Passion Fruit Peel: Composition, Structural Characterization and Emulsion Stability. Foods.

[17] Miller G.L. (1959). Use of Dinitrosalicylic Acid Reagent for Determination of Reducing Sugar. Anal. Chem..

[18] Pattarapisitporn A., Noma S., Klangpetch W., Demura M., Hayashi N. (2024). Extraction of Citrus Pectin Using Pressurized Carbon Dioxide and Production of Its Oligosaccharides. Food Biosci..

[19] Myo H., Nantarat N., Khat-Udomkiri N. (2021). Changes in Bioactive Compounds of Coffee Pulp through Fermentation-Based Biotransformation Using *Lactobacillus Plantarum* TISTR 543 and Its Antioxidant Activities. Fermentation.

[20] Nurcholis W., Sya’bani Putri D.N., Husnawati H., Aisyah S.I., Priosoeryanto B.P. (2021). Total Flavonoid Content and Antioxidant Activity of Ethanol and Ethyl Acetate Extracts from Accessions of *Amomum Compactum* Fruits. Ann. Agric. Sci..

[21] Yakul K., Kaewsalud T., Techapun C., Seesuriyachan P., Jantanasakulwong K., Watanabe M., Takenaka S., Chaiyaso T. (2021). Enzymatic Valorization Process of Yellow Cocoon Waste for Production of Antioxidative Sericin and Fibroin Film. J. Chem. Technol. Biotechnol..

[22] Lacroix I.M.E., Li-Chan E.C.Y. (2012). Dipeptidyl Peptidase-IV Inhibitory Activity of Dairy Protein Hydrolysates. Int. Dairy J..

[23] Seitzer P., Bennett B., Melamud E. (2022). MAVEN2: An Updated Open-Source Mass Spectrometry Exploration Platform. Metabolites.

[24] Herzyk F., Piłakowska-Pietras D., Korzeniowska M. (2024). Supercritical Extraction Techniques for Obtaining Biologically Active Substances from a Variety of Plant Byproducts. Foods.

[25] Radzali S.A., Markom M., Saleh N.M. (2020). Co-Solvent Selection for Supercritical Fluid Extraction (SFE) of Phenolic Compounds from *Labisia pumila*. Molecules.

[26] Apak R., Özyürek M., Güçlü K., Çapanoğlu E. (2016). Antioxidant Activity/Capacity Measurement. 1. Classification, Physicochemical Principles, Mechanisms, and Electron Transfer (ET)-Based. J. Agric. Food Chem..

[27] Kiss A., Papp V.A., Pál A., Prokisch J., Mirani S., Toth B.E., Alshaal T. (2025). Comparative Study on Antioxidant Capacity of Diverse Food Matrices: Applicability, Suitability and Inter-Correlation of Multiple Assays to Assess Polyphenol and Antioxidant Status. Antioxidants.

[28] Singh A.-K., Yadav D., Sharma N., Jin J.-O. (2021). Dipeptidyl Peptidase (DPP)-IV Inhibitors with Antioxidant Potential Isolated from Natural Sources: A Novel Approach for the Management of Diabetes. Pharmaceuticals.

[29] Islam S., Adam Z., Akanda J.H. (2024). Quinic and Caffeic Acids Derivatives: Affecting Antioxidant Capacities and Phenolics Contents of Certain Therapeutic and Specialty Crops Employing Water and Ethanolic Extracts. Food Chem. Adv..

[30] Boulebd H., Carmena-Bargueño M., Pérez-Sánchez H. (2023). Exploring the Antioxidant Properties of Caffeoylquinic and Feruloylquinic Acids: A Computational Study on Hydroperoxyl Radical Scavenging and Xanthine Oxidase Inhibition. Antioxidants.

[31] Balogun F.O., Naidoo K., Aribisala J.O., Pillay C., Sabiu S. (2022). Cheminformatics Identification and Validation of Dipeptidyl Peptidase-IV Modulators from Shikimate Pathway-Derived Phenolic Acids towards Interventive Type-2 Diabetes Therapy. Metabolites.

[32] Alcántara K., Guirao A., Solana-Guilabert A., Agulló V., Díaz-Mula H.M., Valverde J.M., Martínez-Romero D. (2026). Optimizing the Functional Quality of Coffee (*Coffea Arabica* Cv. ’Catidiaf 21′) Pulp: Impact of Cultivation System, Ripening Stage, and Drying Method on Valorization for Food Industry Applications. Appl. Food Res..

[33] Allen K.E., Shahidi F. (2015). Metal Chelators as Antioxidants for Food Preservation. Handbook of Antioxidants for Food Preservation.

[34] Attard T.M., McElroy C.R., Gammons R.J., Slattery J.M., Supanchaiyamat N., Kamei C.L.A., Dolstra O., Trindade L.M., Bruce N.C., McQueen-Mason S.J. (2016). Supercritical CO_2_ Extraction as an Effective Pretreatment Step for Wax Extraction in a *Miscanthus* Biorefinery. ACS Sustain. Chem. Eng..

[35] Wani K.M., Patidar R. (2025). Microwave-Assisted Extraction of Pectin from Lemon Peel Powder: Optimization and Physicochemical Properties. Sustain. Chem. Environ..

