# Supplementary material for: Integrated Green Valorization of Coffee Cherry Pulp Through Bioactive Compound and Pectin-Rich Fraction Recovery for Pectic Oligosaccharide Production
Source: Foods. 2026 Oct 6;15(19):3555. doi: 10.3390/foods15193555 (PMC13665516; doi:10.3390/foods15193555)
Supplement: Supplementary file 1 [file foods-15-03555-s001.zip › Supplementary material.pdf]

Supplementary material

Integrated Green Valorization of Coffee Cherry Pulp through Bioactive Compound and Pectin-Rich Fraction Recovery for Pectic Oligosaccharide Production

Kamon Yakul [1], Tippapha Pisithkul [2], Charin Techapun [1], Chayatip Insomphun 1, Churairat Moukamnerd [1], Phisit Seesuriyachan [1], Wilasinee Jirarat [1], and Thanongsak Chaiyaso [1,*]

[1]Division of Biotechnology, Faculty of Agro-Industry, Chiang Mai University, Chiang Mai 50100, Thailand

[2]Program in Biotechnology, Faculty of Science, Maejo University, Chiang Mai 50290, Thailand

*Correspondence: thanongsak.c@cmu.ac.th

Table S1. The particle size distribution of the CCP powder used in this study.

Sieve mesh size (mm)	Weight (g)	Particle size distribution (%)
1.000	10.41±0.49	2.11
0.600	112.44±5.01	22.77
0.300	189.11±7.51	38.30
0.212	41.16±1.35	8.34
0.150	37.38±2.30	7.57
0.106	29.87±1.63	6.05
0.075	27.59±0.99	5.59
<0.075	45.79±6.96	9.27

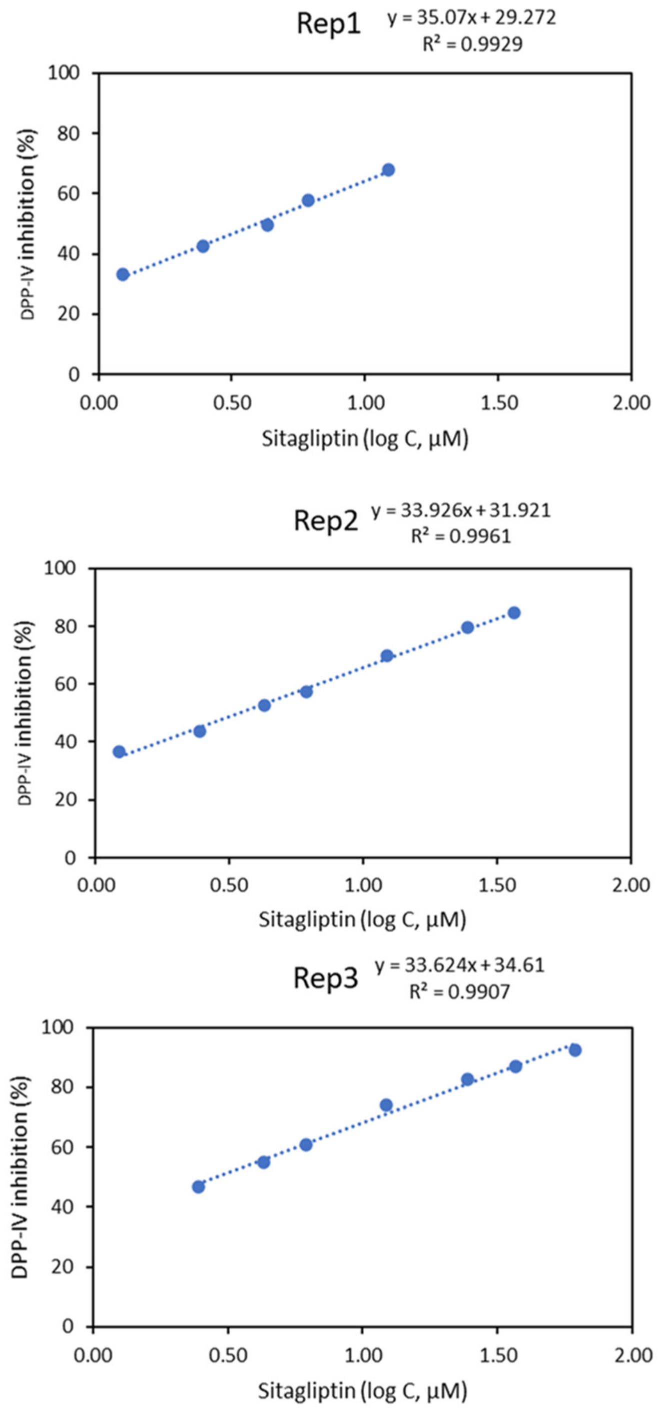

Figure S1. Determination of the IC_{50} value of sitagliptin (SML3205, Sigma-Aldrich) as a positive control for the DPP-IV inhibition assay.

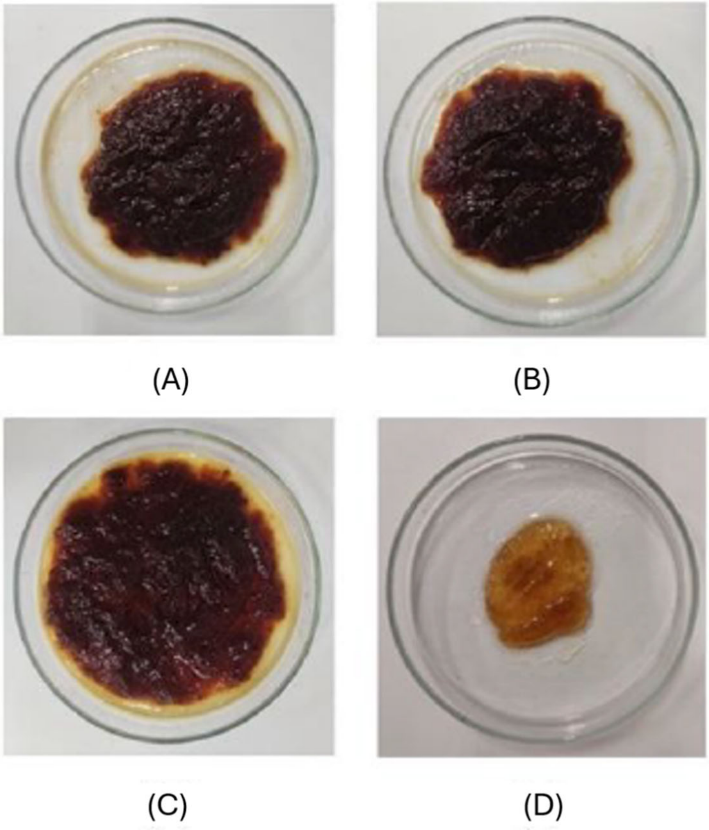

Figure S2. Photographs of pectin-rich fractions recovered from (A) non-treated CCP, (B) SFE-CO_2-treated CCP, (C) ethanol-treated CCP residue, and (D) hot water-treated CCP residue using 2% (w/v) citric acid.
